# Ultrasonic Based Tissue Modelling and Engineering

**DOI:** 10.3390/mi9110594

**Published:** 2018-11-14

**Authors:** Karl Olofsson, Björn Hammarström, Martin Wiklund

**Affiliations:** Department of Applied Physics, KTH Royal Institute of Technology, SE-106 91 Stockholm, Sweden; karlolo@kth.se (K.O.); bham@kth.se (B.H.)

**Keywords:** acoustic trapping, ultrasonic manipulation, tissue engineering, tissue modelling, acoustofluidics, microfluidics

## Abstract

Systems and devices for in vitro tissue modelling and engineering are valuable tools, which combine the strength between the controlled laboratory environment and the complex tissue organization and environment in vivo. Device-based tissue engineering is also a possible avenue for future explant culture in regenerative medicine. The most fundamental requirements on platforms intended for tissue modelling and engineering are their ability to shape and maintain cell aggregates over long-term culture. An emerging technology for tissue shaping and culture is ultrasonic standing wave (USW) particle manipulation, which offers label-free and gentle positioning and aggregation of cells. The pressure nodes defined by the USW, where cells are trapped in most cases, are stable over time and can be both static and dynamic depending on actuation schemes. In this review article, we highlight the potential of USW cell manipulation as a tool for tissue modelling and engineering.

## 1. Introduction

Acoustofluidics has, during the last 1–2 decades, emerged as a straightforward but yet powerful technique for the manipulation of particles and fluids inside micro- and mini-scaled fluid-filled channels and chambers [1]. Traditionally, the great majority of reported acoustofluidic applications utilized standing waves in the MHz-frequency regime, and actuation methods are typically based on either bulk acoustic waves (BAWs) [2] or surface acoustic waves (SAWs) [3]. Another term often used is acoustophoresis, which refers to the technology for manipulating suspended objects in a medium by the use of acoustic fields. The manipulation can relate to levitation [4] and trapping [5], but also to separation and focusing if combined with another force field such as the fluid drag force [6]. If the fluid flow and the standing wave are oriented in orthogonal directions, the term “free-flow acoustophoresis” has been used [7], with a multitude of various applications reported in literature. Not only suspended objects but also the fluid itself can be manipulated, either by the generation of acoustic streaming [8] or in the case of sharp acoustic-contrast gradients in the medium [9].

**Figure 1:**
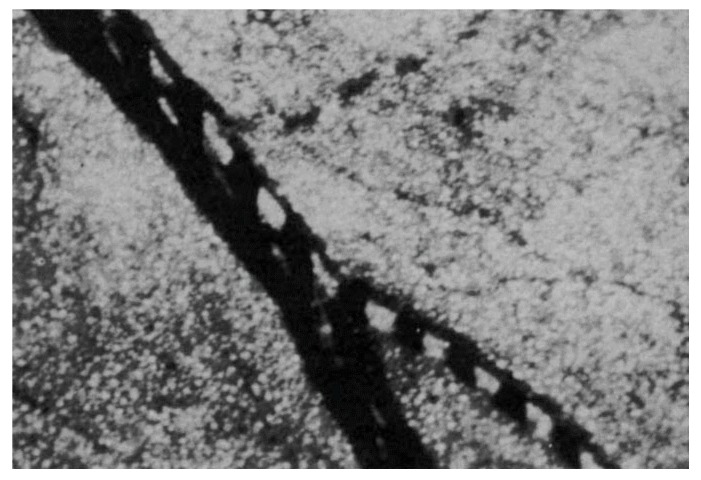
Red blood cell manipulation by ultrasound in a chick embryo. The image shows a chick embryo vasculosa where red blood cells are trapped in clusters separated by half the wavelength at 3 MHz continuous ultrasonic irradiation. The image was acquired through a microscope with 26× magnification. Reprinted with permission from [10].

In parallel with the general development of acoustofluidic technology, the concept of ultrasound-supported tissue engineering and modelling has gradually emerged. However, already in 1971, Dyson et al. showed that red blood cells could be stopped, clumped and patterned by ultrasound in vivo in a chick embryo (Figure 1) [10]. This work has inspired in vitro technology development in the 2000s with studies of cell trapping [11], followed by cell viability studies in standing-wave traps [12,13], and then studies of levitated cell cultures in 2D [14] and 3D [15] for the production of cellular spheroids. Today, ultrasonic standing waves (USWs) have been used for tissue engineering and modelling in various application fields, ranging from tissue explant cultures, tissue modelling, pharmaceutics, cancer studies and immunology. This review article will discuss these applications and the acoustofluidic technology developed and used for the specific purpose of engineering and modelling tissue.

## 2. Cell Functionality in Ultrasonic Standing Wave Traps

To enable ultrasonic trapping-based tissue engineering and modelling, it is of importance to understand the influence of continuous USWs exposure on cell viability, functionality and behavior. It is also important to consider how the cells initially organize when being trapped in a pressure node for predicting tissue shape.

### 2.1. Initital Cell Organization

Initial cell organization, immediately after the start of USW trapping, depends on affinity at cell–cell contact. This is apparent when trapping cells axially in the nodal plane of a one-dimensional standing wave; cells will form either a tightly arranged monolayer or a loosely packed aggregate, depending on contact affinity upon initial cell–cell collision [11]. The initial cell organization also depends on the pressure field, the cell concentration, the occurrence of acoustic streaming, and on the combination of primary and secondary acoustic radiation forces [2,8]. Furthermore, low cell–cell affinity in collisions allows cells to slide over each other and a tight monolayer can be formed where cells arrange hexagonally and thus minimize vacancies and holes in the monolayer. On the other hand, cell–cell collision with high affinity results in cells immediately attaching to each other and loose dendritic aggregates may be formed. Understandably, immediate cell–cell affinity is important for tissue organization because it hints on the cells’ ability to be shaped by the pressure node pattern defined by the resonator design.

### 2.2. Cell–Cell Connections in USW Trapping

Another necessity for tissue formation in USW traps is the opportunity for cells to form stable cell–cell connections while being levitated or retained in USW originating pressure nodes. While the culture time needed for developing stable cell–cell connections is highly cell-dependent, it has been shown that cells trapped in pressure nodes are immobilized rigidly enough for cell–cell networks to be established. However, it has also been shown that the forces involved in cell–cell contacts and cell–substrate contacts dominate over the acoustic radiation forces generated at the pressure levels typically used during USW trapping [16]. This suggests that the forces responsible for cell adhesion and cell organization are primarily of non-acoustic origin. As an example, rat C6 cell line cells trapped in a tight hexagonally arranged monolayer, with an acoustic pressure of 0.27 MPa, showed an increased concentration of the important adhesion molecules NCAM and N-cadherin at the cell–cell interfaces, already after 8 min of USW levitation [17]. Actin filaments in the same C6 monolayers started to accumulate in proximity to the cell–cell interface after 8 min, but a stronger immunofluorescence signal was detected after 30 min of USW trapping. Additionally, primary bovine chondrocytes have been shown to develop functional gap junctions, enabling cell–cell communication, together with connexin-34 and f-actin organization in proximity to the cell–cell interface after 1 h of USW trapping [14].

### 2.3. Cell Behavior after USW Exposure

While these results clearly show that adherent cells are able to form stable and functional cell–cell connections while trapped in an acoustic pressure node, and thus paving the way for USW-based tissue engineering, USW trapping could still alter cell functionality. To investigate whether this is the case or not, Bazou et al. characterized the gene expression of mouse embryonic stem (ES) cells levitated in a monolayer using both high- and low-pressure amplitudes (0.85 MPa and 0.08 MPa, respectively) in a resonator chamber device, where the chamber is a half-wavelength fluid layer in a multilayer resonator [18]. ES cells are strongly affected by the surrounding environment and niche but no statistically significant change in pluripotency, early and late differentiation gene expression could be detected. The retained ES gene expression and pluripotency is strong evidence that USW trapping of cells is minimally invasive and does not affect cell behavior or cell viability, even at acoustic pressure amplitudes up to 1 MPa at a frequency of 2.5 MHz [13,19,20].

### 2.4. Cell Functionality and Behavior in Aggregates

**Figure 2:**
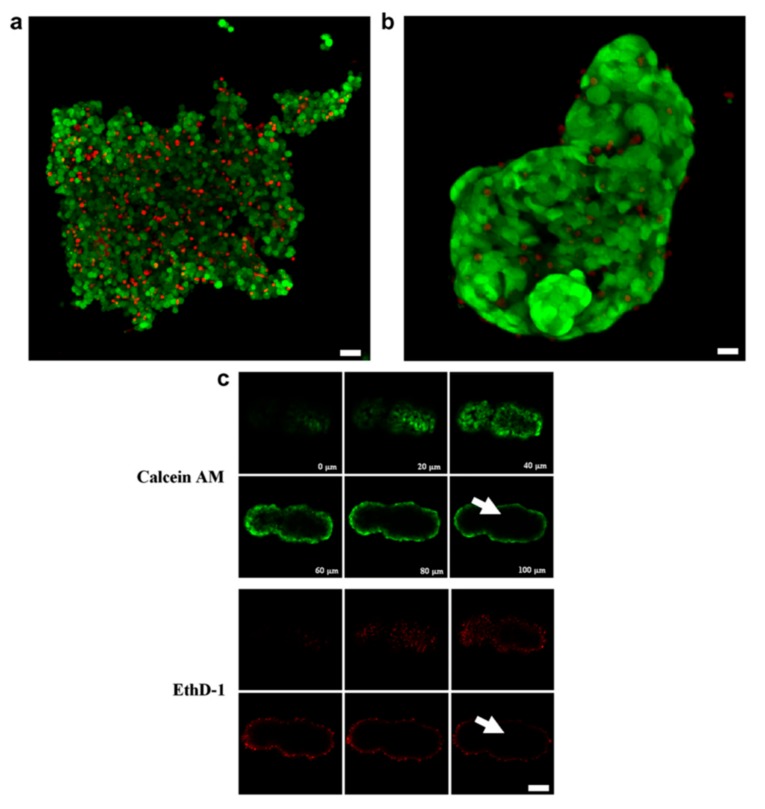
Ultrasound-formed HepG2 cell aggregates embedded in alginate retains viability and forms spheroids over time. Individual HepG2 cells are distinguishable in the initial aggregate (**a**) but after three days in culture membrane spreading and cell–cell connections forms a stable spheroid (**b**). Calcein AM and EthD-1 staining after 10 days in culture shows retained cell viability and poor dye penetration which is typical for larger 3D aggregates (**c**,**d**). Scale bars are 30 µm in (**a**), 10 µm in (**b**) and 20 µm in (**c**). Reprinted with permission from [15].

The step from forming monolayers by USW trapping to culturing 3D cell aggregates is not big. In the device where most of the above-mentioned studies were performed, the third dimension is added to the already established 2D monolayers by increasing the cell concentration until multiple layers are trapped in contact with each other [15]. To demonstrate this, aggregates of trapped HepG2 hepatocellular carcinoma cell line cells were stable enough to be handled after 5 min of USW trapping and could be transferred and encapsulated in alginate. The encapsulated aggregates showed retained cell viability over 10 days of culture compared to non-encapsulated 3D HepG2 aggregates (Figure 2). Confocal microscopy images of aggregates stained with calcein AM and EthD-1 revealed poor dye penetration into the 3D aggregates, which indicates mature cell–cell contacts and thus limited molecule diffusion (Figure 2c). Additionally, F-actin staining showed similar actin organization to that in the monolayer case described above but with the added third dimension to the cell aggregates [21].

## 3. Cell Patterning and Tissue Shaping

The retained cell viability, function and behavior in USW traps enables ultrasound-based tissue engineering where one of the main advantages of acoustophoresis can be exploited; label-free and gentle cell positioning with micrometer precision in patterns which are flexible in terms of appearance. Depending on how the resonator chamber and transducer placement are designed, pressure node patterns with a high degree of customization can be achieved.

### 3.1. Single Pressure Node

Depending on whether a single or several pressure nodes are desired for an application, the choice of resonator chamber dimensions, together with transducer actuation frequency, plays a vital role. There are many examples of devices where a single pressure node defines the tissue shape. One of the earliest devices where cell trapping was performed, developed by Coakley et al., relied on a single transducer in contact with a resonator chamber with a height corresponding to the half wavelength of the introduced acoustic wave [22]. A glass reflector induced a standing wave and the resulting pressure field was strong enough to trap and continuously levitate cells. This device is a classical layered resonator where a matching layer, typically with a thickness of quarter of the wavelength, is used to optimize pressure intensity transmission into the resonator chamber carrying the suspended particles or cells to be trapped. A reflection layer with an optimal thickness of an odd multiple of a quarter of the wavelength is used to retain the pressure inside the resonator chamber [6]. Similar layered resonator designs have also been developed to produce cartilage explants [23] and are described extensively below. Furthermore, single pressure node systems have also been used for producing bacterial agglomerates and biofilms [24,25,26,27], primarily for biosensing applications [28].

### 3.2. Multiple Pressure Nodes

**Figure 3:**
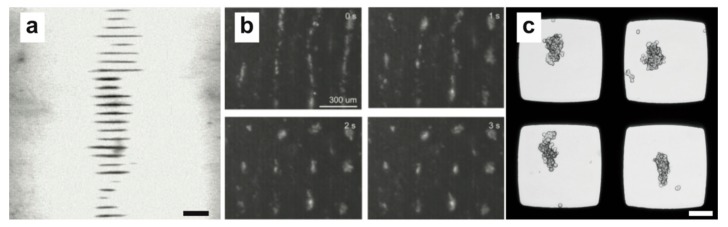
Multiple acoustic pressure nodes are an effective way to parallelize tissue formation. Numerous pressure nodes can be obtained by using multiple wavelengths in a single resonator chamber (**a**) (scale bar: 1 mm) [29], producing pressure nodes arrays through orthogonal one-dimensional pressure fields (**b**) [30] or using multiple microwells where the half-wavelength (λ/2) criterion is met in two dimensions (scale bar: 100 µm) (**c**). Reprinted with permission from (**a**) [29], (**b**) [30], and in (**c**) experiment by Karl Olofsson.

A facile approach to creating multiple pressure nodes to parallelize cell aggregate formation is to use a similar layered resonator device described above, but tune the frequency or resonator dimensions to fit multiple wavelengths in the fluid layer. This effectively initiates multiple pressure nodes for cells to be trapped in and has been implemented by Liu et al, where multiple disk-shaped HepG2 hepatocellular carcinoma cell aggregates (Figure 3a) were formed along the axis of the wave propagation [29]. These HepG2 cell aggregates were formed during 30 min of continuous USW trapping before being transferred to a low-attachment substrate for further culture.

One possible issue with using the USW along a single axis is that cells will be trapped in a nodal plane and thus the final cell agglomerate will be disk-shaped. If a spherical-shaped cell aggregate is desired, two horizontally oriented one-dimensional pressure fields perpendicular to each other will by superposition yield a pressure node of circular shape. This can be accomplished in several ways and even in a multiple wavelength fashion, where an array of circular pressure nodes can be achieved in a shared resonator chamber. One such device was beautifully presented by Nield et al., where two piezo strips perpendicular to each other could individually establish a multiple-wavelength one-dimensional pressure field in a resonator chamber while a pressure node array was introduced by simultaneously actuating both piezo strips [30,31]. Individual or multiple breast epithelial MCF10A cells were readily trapped with a half-wavelength distance (Figure 3b). Here, the third trapping dimension is added by the gravity to the other two dimensions of the force field controlled by the acoustic field. Surface acoustic waves have also been used to define a similar horizontal pressure node array in a PDMS chamber. The pressure fields from two pairs of interdigital transducers (IDTs) aligned orthogonally were able to trap and maintain HepG2 cell aggregates for 30 min before extraction and further culture off-chip [32]. A neat feature with this device is that cell aggregate levitation was achieved without radiation forces by small micro vortexes surrounding the pressure node, which prevented cells from interacting with the substrate during cell aggregate formation and thereby facilitating easier cell aggregate withdrawal.

Another approach to obtaining circular pressure nodes has been developed by Wiklund and co-workers for parallel horizontal 2D positioning of cells in multiple microwells. In this technique, the microwells act as small resonator chambers where the half-wavelength criterion is fulfilled in two dimensions and efficiently creates a single pressure node in the middle of the well [33]. The developed platform consists of a multi-well microplate with 100 microwells etched through a silicon wafer and bonded to a glass plate. When actuated by a transducer, a single symmetric pressure node is formed in all 100 microwells simultaneously. These microwells have been used to study cell positioning, synapse formation by natural killer (NK) cells and NK cell–tumor model dynamics [16,34,35,36]. A key challenge when using multiple microwells sharing resonator material is the difficulty to predict pressure-node shape due to complex resonances and acoustic crosstalk between the wells. During single frequency actuation of the multi-well microplate, suspended cells or particles typically arrange in bands with a major axis orientation that changes with frequency [34]. Circular pressure nodes, suitable for e.g., spheroid formation (Figure 3c), can be achieved in the microwells by linearly sweeping the actuation frequency around a central frequency of ±4% (typically) and thus averaging all line-shaped pressure nodes into the common intersection which coincides with the microwell center.

### 3.3. Pressure Node Patterns

There are plenty of opportunities for USW-based cell patterning on surfaces by creative chamber designs and transducer placements. One device, where several pressure node patterns can be achieved on demand, has been developed by Bernassau et al., where piezo transducers constitute the walls in a resonator chamber [37]. More specifically, seven piezos were attached to a circuit board and arranged to a heptagon before being mounted on a glass cover slip and thus created a basin where cells could be trapped. Depending on which of the seven transducers that were actuated, both line and hexagonal trapping patterns could be created. Typically, the thickness mode actuation of two transducers introduced line patterns while hexagonal patterns arose when actuating three piezos simultaneously. These patterns could also be relocated by phase shifting of the transducers by 180 degrees, which introduced a simple way of assigning different cell types to separate locations by adding cells sequentially before and after the phase shift. This technique has matured and is now able to precisely position on-demand particles in 3D with a user-defined particle separation [38,39].

Line-shaped pressure node patterns on a substrate have also been presented in a device based on surface acoustic waves [40]. A pair of IDTs, with a period of 300 µm, were deposited on a LiNbO_3_ substrate with a bonded PDMS channel and used to arrange an initial line pattern of cells. After the first batch of cells had attached to the substrate, second cell line was seeded into the device while tuning the relative phase of signal to the two IDTs by 180 degrees, which displaced the pressure node lines by a quarter of a wavelength. As a proof of principle, initial separation HeLa cells and HUVEC cells to different lines were used to measure cell migration.

Another strategy is to generate multiple-node cavity resonances for producing cell patterns similar to Chladni figures [41]. This has been used by Demirci et al. in a device based on a square resonator plate with a liquid chamber coupled to a vibration generator [42]. A multitude of Chladni figures are achievable when driving the vibration generator at resonance frequencies (50–200 Hz) and have been demonstrated to be compatible with cellular spheroids [43] and human iPS cell derived cardiomyocytes [44] in a hydrogel precursor liquid which stabilized the pattern when settling. An intriguing prospect with this audible-frequency approach for acoustic positioning in tissue engineering is the scalability; over 10^4^ spheroids could be assembled within 15 s.

### 3.4. Acoustic Streaming

One slightly overlooked effect of ultrasonic manipulation in terms of cell positioning is acoustic streaming. There are several mechanisms driving acoustic streaming and everything from micro-vortexes to larger bulk streaming can be observed [8]. While this phenomenon can be problematic in many USW-based trapping devices, where the drag force from the acoustic streaming may counteract the radiation forces [45], it can also be of great use if exploited correctly. As an example, cell manipulation has been achieved by acoustically driven micro-vortexes from both sharp-edge features [46] and microbubbles in microfluidic channels [47].

Acoustic streaming can also be directly used for cell aggregation and tissue engineering, which was demonstrated by Friend and co-workers [48]. By using a standard plastic 24-well plate, a fluid couplant and a piezo displaced and tilted 20° relative to the wellplate bottom center, bulk acoustic streaming was generated. The fluid flow in this setup followed the circular well shape and BT-474 breast cancer cells seeded in the well were concentrated in the well center and over time formed a cell agglomerate which resembled a spheroid. An intermittent actuation scheme of the piezo (3 s on every 1 min for 72 h) driving the acoustic streaming in a low cell attachment well ensured a good spheroid formation while keeping a steady temperature and retained cell viability.

### 3.5. Arbitrary Pressure Field by Acoustic Holography

While all the above-mentioned devices have been proven to robustly trap, organize and shape cell aggregates into tissue-mimicking pieces during continuous culture, they are restricted in terms of possible achievable pressure node shapes. The available shapes possible to create in described devices is more than enough for most purposes, but new exciting applications could be developed if arbitrary pressure node shapes could be defined. We strongly believe that the acoustic holography technique recently developed by Peer Fischer’s lab could be an avenue for ultrasonic tissue engineering, where any possible cell aggregation shapes can be obtained [49,50].

**Figure 4:**
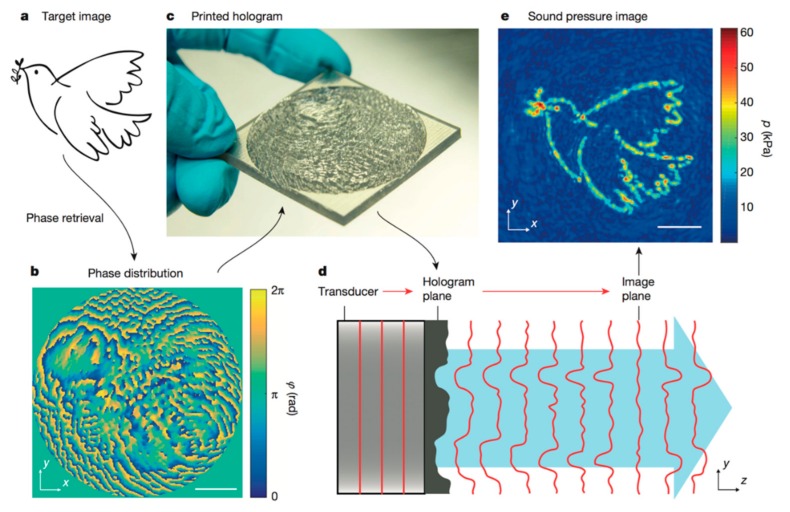
Acoustic holography can be used to form arbitrary pressure field images. The target image (**a**) is used to backward calculate the required phase distribution (**b**). The 3D printed transmission hologram, as a height topography in a phase plate (**c**), is placed in front of the transducer (**d**) to acquire the sound pressure image (**e**). Scale bar: 10 mm. Reprinted with permission from [49].

The acoustic holography technique is based on adjusting the relative phase of every point in planar wave fronts which is due to the fact that constructive interference defines an arbitrary pressure field at a specified distance along the propagation axis. In the first published study [49], the wave front phase shift was decided by the topography of a 3D-printed plastic phase plate placed in front of the transducer (Figure 4). The topography thickness introduced the relative phase shift between points in the wave front because of a speed of sound difference compared to the chamber fluid. The topography was determined by taking the desired pressure amplitude field as input to an iterative angular spectrum approach algorithm, where the needed phase shift is backwardly calculated.

This technique was beautifully illustrated by manipulating PDMS microbeads, which have a negative contrast factor and thus accumulate to the pressure anti-node, into shapes not possible to acquire with a standard resonator chamber design [50]. Due to the holographic redundancy principle, another very intriguing characteristic of this technique is the ability to define multiple 2D arbitrary pressure fields along the propagating axis. This would potentially allow multiple cell aggregates of different shapes to be cultured in the same bioreactor.

While this technique is very interesting for future applications within the ultrasonic trapping-based tissue modelling and engineering field, there are still some issues that have to be addressed. Firstly, the pressure field feature sizes presented in the initial publications might be too large for tissue engineering with cellular precision. Nutrient and gas gradients in large, non-vascularized cell aggregation elements affect both cell viability and function. The smallest acoustically resolvable feature size in the pressure image is half wavelength (λ/2), which for 2 MHz is ~0.4 mm in water, and therefore depends on transducer frequency and manufacturing resolution of the phase plate topography encoding the phase shift. Since high-frequency transducers are available, manufacturing development of the phase defining plate details might be needed.

Secondly, the pressure amplitude fields achieved in the publication might not be enough to trap and maintain particles and cells with a positive contrast factor. A pressure amplitude field meant for particles with a positive contrast factor, where the trapping configuration is defined by pressure nodes, is less intensity-efficient compared to a positive pressure field image for manipulating particles with a negative contrast factor, which was shown in the pioneering studies [50]. If it would be possible to combine holographic techniques with a standing wave setup, energy will be retained in the system and we believe that higher transducer frequencies and finer phase plate topographies, combined with a standing wave setup, might be efficient enough to robustly trap cells in arbitrary shapes without generating unfeasible amounts of heat.

## 4. Tissue Modelling and Engineering

To induce tissue-like properties in a cell culture within a microsystem, the physiochemical microenvironment is of key importance. Control over factors, such as chemical ques, cell–cell interactions by proximity to other cells, mechanical stimulation, as well as perfusion and temperature, may have to be maintained for days in order to induce a desired behavior in a particular cell culture. These challenges are similar for both tissue engineering and tissue modeling applications where the aim is to create small-scale tissue models such as 3D cultures, spheroids, and organs-on-chips.

While small-scale tissue models have a great potential to advance our understanding of biological systems and evaluate therapeutics, tissue engineering offers the prospect of generating tissue in a bioreactor that can then be used as an explant for a patient. Since the control over cellular microenvironment offered by a microsystem may be required to trigger tissue formation, a major challenge is to generate large enough tissues. Understandably, the required size is determined by the type of tissue that is to be generated. While there may be applications where an mm-sized piece of tissue can make a difference, it is clear that if tissues could be made one or two orders of magnitude larger, the impact would be much wider. Of practical importance is also that the generated tissue needs to be large enough for a surgeon to handle and that it can be extracted from the chip.

A large amount of biomaterials research has been directed to creating support structures or scaffolds that can create suitable 3D patterns of cells to be used for tissue engineering [51]. This approach is promising as it can create precise arrangements of cells in a structure with microscale features that repeats itself over large distances. Many materials can be explored as scaffolds for tissue engineering but hydrogels are perhaps one of the most promising candidates [52]. Crucially, a patterned hydrogel can make it possible to embed cells in a material that allows for chemical diffusion.

During culture, the cells and any used scaffold become intertwined. If used as an explant directly, meaning that exogenous materials are introduced into the body, care has to be taken in order to find a material that will integrate well or can be completely dissolved while maintaining tissue integrity. Challenges also occur in terms of functionalization where the scaffold material has to be suitably functionalized in order for cells to grow on it. Limited diffusion can also be a major issue when aiming for larger pieces of tissue, where structures that do not allow for sufficient oxygen diffusion can produce necrotic regions. Together, these challenges introduce a lot of demands on the scaffold material.

### 4.1. Scaffold-Free Approach

**Figure 5:**
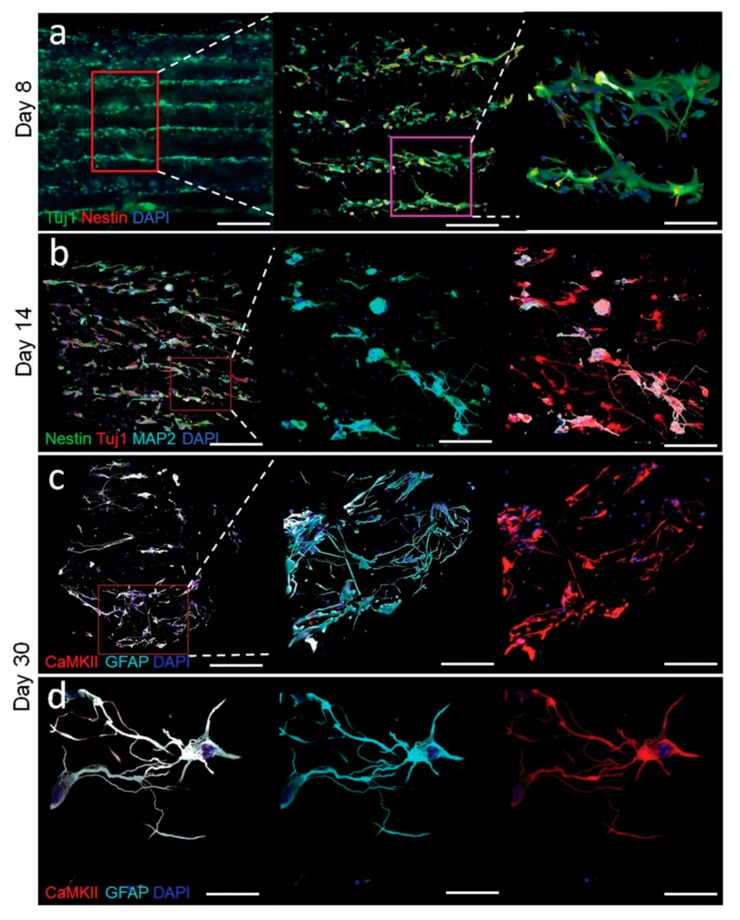
Patterned and in situ differentiated neurons can model the cerebral cortex. USW-based arrangement of neural progenitor cells during fibrin hydrogel cross-linking formed stable cell layers embedded in the hydrogel suitable for long-term culture without USW. This allowed for differentiation of the neural progenitor cells into neurons in situ which interacted between layers after 8 days (**a**). Fourteen days after culture, mature neuron maker MAP2 was co-expressed together with Tuj1 (**b**). The excitatory marker CaMKII and glial cell marker GFAP are detected after 30 days of culture (**c**,**d**). All scale bars indicate 30 µm. Reprinted with permission from [53].

Acoustic particle manipulation can offer a way to sidestep some of these challenges by working towards scaffold-free tissue engineering bioreactors. As discussed previously, ultrasound has the capability to arrange cells in patterns with micrometer features over extended areas [37]. By generating sparse arrangements of cells, an improved diffusion rate could be accomplished since no scaffold material is present. Even in comparison with a patterned hydrogel, improvements could be made by allowing higher diffusion constants and the possibility to perfuse the entire volume with flow. Bouyer et al. demonstrated a beautiful setup where neural tissue is generated in a sparse arrangement of human stem cell-derived neural progenitor cells assembled in layers and stabilized in a fibrin hydrogel by means of ultrasound [53]. After initial ultrasound pattering and stabilization in the fibrin hydrogel, the neural progenitor cells differentiated in situ and in absence of ultrasound into neural cells by culturing the embedded cell layers in differentiation media. These neural cells showed interaction between layers and thus mimicked the cerebral cortex (Figure 5). The work also showcased that since cells are attracted to the pressure nodes of the standing wave, the spacing between cell aggregates can be tuned by the frequency of the ultrasound (λ=cf). However, a scaffold-free approach to tissue engineering based on acoustofluidics in its current form comes with two foreseeable limitations; within the pressure node, cells will be brought into immediate proximity due to force gradients and secondary forces; and the absence of a substrate may be a problem for some cell types. Whether or not these points will be adverse or beneficial to the quality of the generated tissue will likely depend on cell types and the type of tissue that is engineered.

### 4.2. Tumor Modelling by Spheroid Culture

An example of where the benefits of scaffold-free tissue culturing are evident is solid tumor modelling. In solid tumors, the microenvironments in and surrounding the tumors are known to influence immunological and therapeutic response [54]. Multicellular tumor spheroids (MCTSs), which are 3D cultured spherical tumor cell aggregates, are appreciated as tumor models since they reproduce the tumor microenvironment through cell–cell contacts, biochemical and biophysical cues [55]. There are many methods around for MCTS production [56]. Compared to such alternative methods for culturing MCTSs, USWs is an attractive approach since ultrasonic radiation forces are gentle, and non-invasive while still offering a high degree of precision in terms of MCTS size and position due to the possibility to control both the direction and magnitude of these forces. Other benefits of USW-based spheroid cultures include good compatibility with high-resolution microscopic imaging techniques, and the possibility to culture in both 2D and 3D using the same platform [57,58]. The acoustic radiation forces in USW-based spheroid cultures function in a similar way as the gravitational forces in, for instance, hanging drop cultures, but with the added flexibility of being able to dynamically control both the magnitude and direction of the force field. The challenge with USW-based techniques, compared to conventional spheroid formation techniques, is the higher technical threshold, such as managing heat produced in the piezoelectric material and carefully designing resonant structures.

The most efficient way to produce multiple MCTSs is to use an array of circularly defined pressure nodes. An SAW-based device described above was used to initiate cell aggregates in a shared reservoir for later extraction and maturation into MCTS off-chip [32,59]. HepG2 hepatocellular carcinoma cell line was used to show parallel formation of 150 equally sized cell aggregates that were kept levitated and trapped for 30 min before being extracted. Around 50 out of 150 cell aggregates could repeatedly be extracted for further culture in standard Petri dishes. As a proof of principle, a drug sensitivity experiment with anti-cancer drug 5-fluorouracil was used to show decreased drug sensitivity in the MCTSs compared to a regular 2D culture.

BAWs can also be used to introduce a pressure node array in a resonator chamber for MCTS production [31]. MCTS formation is also one of the focuses in our group where we use a multi-well microplate which is described in detail above. In the first publication where this multi-well microplate was demonstrated to culture tumor models, HepG2 hepatocellular carcinoma was used to show on-chip formation, staining and analysis of tumor models [35]. The tumor models were formed by seeding a single cell suspension which settled in the microwells before the microplate was mounted onto the transducer. The frequency-modulated actuation of the transducer introduced a single circularly shaped pressure node in all microwell centers, in which the cells were trapped into an aggregate. Investigations on required USW trapping times for stable 3D cell aggregate formation showed that 48 h was sufficient. Since the microwell bottoms are made out of untreated glass, the cell aggregates were able to interact and attach themselves to the bottom substrate, and thus an anchored hemispherical tumor model was cultured. These tumor models were used to study the dynamics and potency of NK cells against solid 3D tumors.

One issue with this setup when using other cell lines rather than HepG2 to culture tumor models is that, instead of forming hemispherical 3D tumor models, the cells migrate and form a 2D culture when kept in a regular incubator after USW exposure. To overcome this issue and enable more solid tumor types to be cultured in the platform, a protein repellent polymer coating [60] was applied to the multi-well microplate to remove interaction possibilities between the cell aggregate and substrate. The pressure field magnitude originating from USW was shown not to be influenced by the thin (~20 nm) polymer coating and the resulting MCTS was of high quality. All cell lines tested, including HepG2 hepatocellular carcinoma, A498 and ACHN renal carcinoma, and LUTC-2 thyroid carcinoma, did form stable MCTSs after 24 h of USW trapping and were able to be continuously cultured on-chip in a standard incubator [57].

**Figure 6:**
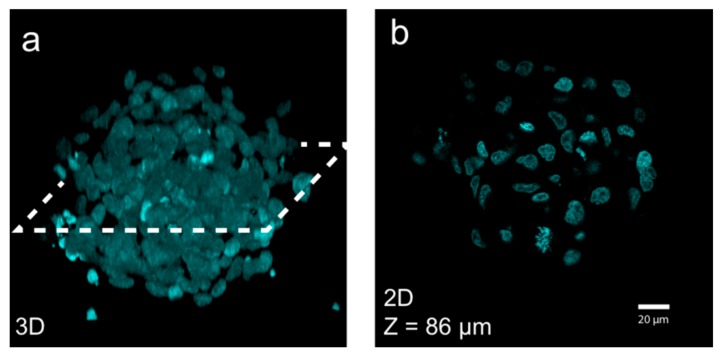
On-chip confocal imaging illustrates the strength with USW-based multi-cellular tumor spheroid (MCTS) culture platforms. Nuclear dye DAPI-stained A498 MCTSs mounted in a refractive-index-matching solution allowed for 3D reconstruction (**a**) from a stack of optical sections (**b**) acquired by on-chip confocal microscopy. The whole MCTS volume could be imaged in detail. Figure adapted from [58].

While there are many devices and techniques that aim at culturing MCTSs, USW-based MCTS culture platforms have some advantages when designed properly. One of the main methods of MCTS characterization and analysis is based on optical microscopy. However, glass, a material with superb optical quality, is rarely used as a substrate in MCTS culture setups. In the acoustofluidic field, glass is, due to its good acoustical properties, an established material component, and most acoustphoretic devices are therefore compatible with high-quality imaging [61]. Especially glass of a cover glass thickness (~170–200 µm), which is often considered in the optical design of microscopy objectives, is an ideal material choice in devices when aiming for high-quality on-chip microscopy. As an example, the glass bottom of the multi-well microplate is of cover glass thickness [62] and both good time-lapse and 3D confocal imaging (Figure 6) can be achieved if mounting the spheroid in a refractive-index-matching solution [35,57].

### 4.3. Tissue-Engineered Cartilage Explants

**Figure 7:**
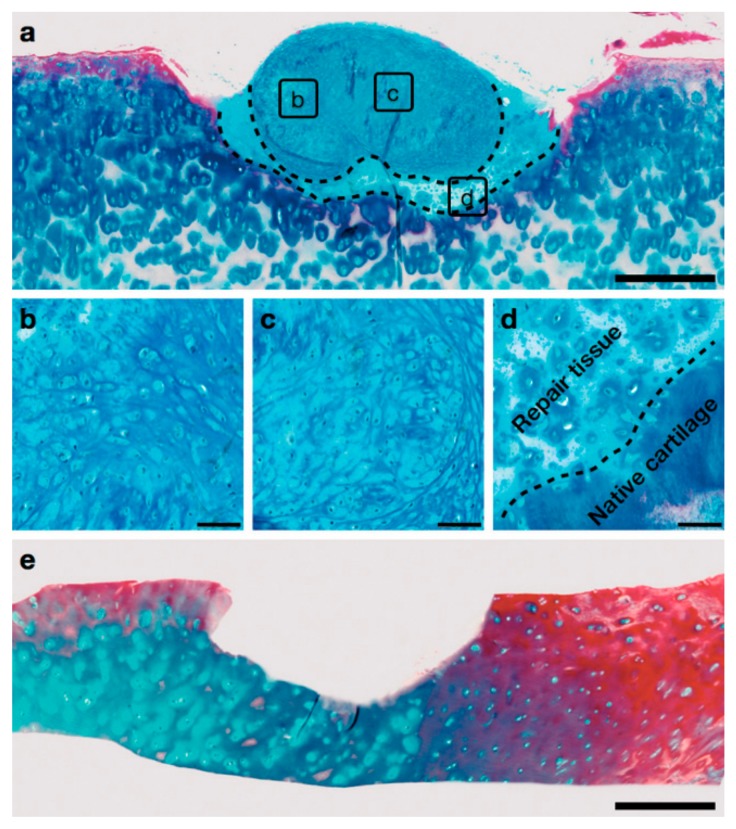
Implantation of an acoustically generated cartilage graft in healthy human tissue with a drilled defect (**a**). Close-ups are shown in (**b**,**d**), and in particular, (**d**) shows the fusion between the native tissue and the graft with formation of hyaline cartilage in the gap. A defect before implantation is shown in (**e**). The scale bars in (**a**,**e**) correspond to 500 µm; whereas the scale bars in (**b**,**d**) correspond to 50 µm. Reprinted from [63] published by the Royal Society of Chemistry.

In addition to tumor models, other types of healthy tissues have been engineered with USWs. One such successful example is the generation of human cartilage explants [63]. This work targets the osteoarthritis a condition that includes the formation of small lesions away from the bone marrow. The absence of marrow means that natural promotion of repair will not occur before the lesions have grown significantly in size. The authors present an approach where millimeter-sized explants are produced in an acoustofluidic bioreactor and could potentially be used to stop growth of the lesion. Two devices for generating suitable explants have been presented: (1) a system based on glass capillary acoustic trapping with perfusion and (2) a passive submersible system based on coverslips and polymer spacers. Both systems are able to generate neocartilage grafts. Figure 7 shows fusion of a cartilage graft taken from the capillary device 16 weeks after implantation in a created defect in host human articular cartilage.

To produce the neocartilage grafts, human articular chondrocytes (HACs) were isolated from osteoarthritic patient samples. Levitated aggregates of isolated HACs were then formed and cultured under acoustic levitation for a period of 21 days to form the grafts. The timescales involved before a cell culture starts to exhibit tissue properties puts great demands on robustness of the bioreactor. One can realize that if there is an incident, such as the formation of a bubble, a software or equipment malfunction, or even a power outage, up to three weeks of culture may be lost. It may also be hard to take a mass-parallelization approach since patient samples can be rare and a large aggregate is desired. In the second paper from the same group, parallelization of four devices in combination with a reductionist approach, where the entire fluidic system has been removed, seems to have been used to tackle this issue [23].

In devices where automatic frequency tracking [64] is not possible, actuation using frequency sweeps is increasingly being applied. This allows for sacrificing acoustic force magnitude in order to achieve robustness against temperature variations and the opportunity to parallelize devices with slightly different resonance frequency using a single driving unit. Typically, the frequency sweep is roughly centered around the resonance frequency moving linearly from a start frequency to a stop frequency with a certain sweep rate or sweep period. Deciding the bandwidth determines the trade-off between time spent driving active modes (where particle manipulation is accomplished), and the variations in resonance frequency (e.g., due to temperature variations or device dissimilarities) that can be accommodated.

**Figure 8:**
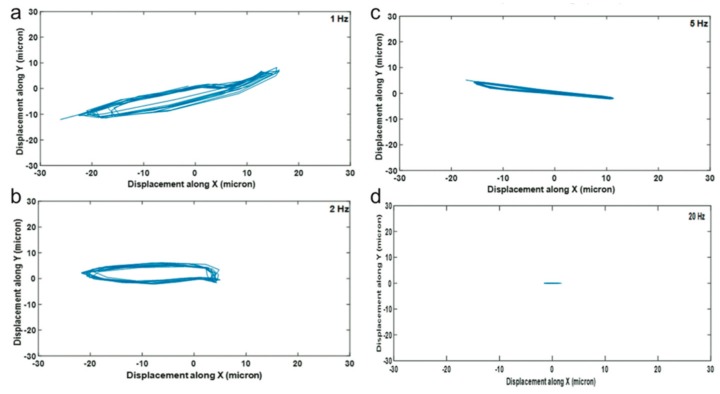
Actuating the acoustic trap with a frequency sweep will move a levitated chondrocyte cell aggregate laterally. The displacement can be controlled by the sweep rate, providing a means to control the mechanical stimulation during tissue formation. In the figure, the aggregate position along *x* and *y* directions over time is measured at the sweep rates 1 Hz (**a**), 2 Hz (**b**), 5 Hz (**c**) and 20 Hz (**d**) Reprinted with permission from [23] published by the Royal Society of Chemistry.

For the generation of neocartilage grafts, frequency sweeps are being used to enable actuation of four devices with a single unit, but more importantly in order to provide mechanical stimulation without a fluid flow. As each frequency within the sweep bandwidth will produce a slightly different acoustic field, for instance, due to low-frequency lateral resonances, the position with lowest acoustic force potential can alternate during the course of the sweep. Figure 8 shows a measurement of the lateral position during the course of several frequency sweeps for a set of sweep rates. As can be seen, a larger displacement cycle is obtained for lower rates. Applied shear will, in the end, not be determined by the displacement but by the speed at which the aggregate moves. The speed is in turn determined by the factors displacement, sweep period, driving amplitude, and bandwidth taken together. Interestingly, using histology, the authors in [23] compared 2-Hz and 50-Hz sweep rates. They concluded that the higher mechanical stimulation found at 2 Hz was advantageous for the formation of cartilage explants.

### 4.4. Acoustic Hydrogel Hybrids

A potential avenue of further exploiting ultrasound for tissue engineering is the combination of acoustic trapping and hydrogels. The possibility to successfully encapsulate constructs generated in an acoustic bioreactor in hydrogels was demonstrated by Bazou et al. [15] in 2008 and is described in the cell functionality section above. In light of the new developments towards tissue engineering, similar results have recently been obtained where the combination of ultrasound and hydrogels is highlighted as a way forward in tissue engineering [53,65,66,67,68,69,70,71]. These studies highlight the potential to apply this approach in a tissue engineering context and that similar results can be achieved using either SAW or BAW technology.

Whether there is a distinct advantage to pattern cells using acoustics, instead of the superior spatial control granted by a patterned hydrogel, remains to be seen. Perhaps when more is known about how to generate tissue models by ultrasound, some of these developments can be translated to tissue engineering by the use of gels. For instance, if the use of controlled mechanical stimulation by positional changes is needed only in the early stages of culture, current literature suggests that it should be successfully combined with a passive hydrogel for long-term culture.

### 4.5. Possibilities for Clinical Use

Most of the devices described in this review are tools for modelling tissue in basic research or potentially for drug screening, and thus have unknown applicability in various clinical settings. However, while we are not aware of any device for tissue engineering which has received regulatory approval for use in living subjects, there are potentially benefits to be aware of in some of the concepts reviewed here. As an example, USW-based trapping might bridge the scaffold-free and scaffold-based tissue engineering approaches and gain synergistic effects [72]. One main disadvantage with scaffold-based tissue engineering is low seeding density interfering with cell organization and self-assembly. For many tissues, including bone, cardiac muscles and liver, cell arrangement is critical for cell–cell communication and thus tissue development and function [73]. If a scaffold is required for, e.g., mechanical stability and biofunctionalization in regenerative medicine, we believe that low seeding density issues can be addressed by utilizing USW-based manipulation for patterning and cell concentration before scaffold cross-linking [70]. This approach has been investigated by Utkan Demircis’ lab for cell-loaded hydrogels [74], spheroids [75], human iPS cell-derived cardiomyocytes [44] and neural progenitor cells [53]. These cultures were suspended in a fibrinogen solution and assembled in patterns before the fibrinogen cross-linking. After complete fibrinogen cross-linking, the construct can be removed and cultured passively which gives this technique a fairly high throughput. The strength of this technique was shown with a 3D cardiac tissue construct where cardiomyocytes showed better synchronization and contraction-relaxation rate when assembled and concentrated in an acoustically defined pattern.

An SAW-based technique has also been used to spatially pattern and concentrate cells in a polymer solution flowing in a glass capillary before polymer cross-linking by UV light [65]. The cured polymer was extracted from the device as a fiber which could potentially be used for building larger tissue complexes.

While the above-mentioned studies highlight the potential benefits of using USW-based trapping in regenerative medicine and tissue engineering, scalability and throughput have to be addressed for future clinical implementations.

## 5. Conclusions

Since the advent of implementing USWs for the manipulation of cell position, the technique has matured into prolonged trapping of cells in pressure nodes for modeling and engineering tissue. The current state of art allows for various tissue modeling such as tumor spheroid culture and tissue engineering of cartilage explants in dynamic bioreactors. Intriguingly, many of the USW-based tissue engineering devices developed are microscaled and flow-through-based, which allows for many microfluidic techniques to be combined with the USW bioreactors, and thus opens up perfusion and in situ analysis possibilities. Additionally, future possibilities to achieve arbitrary pressure nodes shaped, maybe even in three dimensions, through acoustic holography, might further establish high-frequency acoustics as a possible advanced scaffold-free tissue engineering approach. We believe that the USW-based tissue engineering field has yet to reach its full potential.
